# *Plasmodium falciparum* Rab5B Is an N-Terminally Myristoylated Rab GTPase That Is Targeted to the Parasite's Plasma and Food Vacuole Membranes

**DOI:** 10.1371/journal.pone.0087695

**Published:** 2014-02-03

**Authors:** Carinne Ndjembo Ezougou, Fathia Ben-Rached, David K. Moss, Jing-wen Lin, Sally Black, Ellen Knuepfer, Judith L. Green, Shahid M. Khan, Amitabha Mukhopadhyay, Chris J. Janse, Isabelle Coppens, Hélène Yera, Anthony A. Holder, Gordon Langsley

**Affiliations:** 1 Laboratoire de Biologie Cellulaire Comparative des Apicomplexes, Département d'Immunologie, Inflammation et Infection, Faculté de Médicine, Université Paris Descartes - Sorbonne Paris Cité, Paris, France; 2 Institut National de Recherche Médicale U1016, Centre National Recherche Scientifique UMR8104, Cochin Institute, Paris, France; 3 Parasitology, Center of Infectious Diseases, Leiden University Medical Center, Leiden, The Netherlands; 4 Division of Parasitology, MRC National Institute for Medical Research, Mill Hill, London, United Kingdom; 5 National Institute of Immunology, Aruna Asaf Ali Marg, New Delhi, India; 6 Department of Molecular Microbiology and Immunology, Johns Hopkins University Bloomberg School of Public Health, Baltimore, Maryland, United States of America; Ehime University, Japan

## Abstract

*Plasmodium falciparum* (Pf) has a family of 11 Rab GTPases to regulate its vesicular transport. However, PfRab5B is unique in lacking a C-terminal geranyl-geranylation motif, while having N-terminal palmitoylation and myristoylation motifs. We show that the N-terminal glycine is required for PfRab5B myristoylation *in vitro* and when an N-terminal PfRab5B fragment possessing both acylation motifs is fused to GFP and expressed in transgenic *P. falciparum* parasites, the chimeric PfRab5B protein localizes to the plasma membrane. Upon substitution of the modified glycine by alanine the staining becomes diffuse and GFP is found in soluble subcellular fractions. Immuno-electron microscopy shows endogenous PfRab5B decorating the parasite's plasma and food vacuole membranes. Using reverse genetics *rab5b* couldn't be deleted from the haploid genome of asexual blood stage *P. berghei* parasites. The failure of PbRab5A or PbRab5C to complement for loss of PbRab5B function indicates non-overlapping roles for the three *Plasmodium* Rab5s, with PfRab5B involved in trafficking MSP1 to the food vacuole membrane and CK1 to the plasma membrane. We discuss similarities between *Plasmodium* Rab5B and *Arabidopsis thaliana* ARA6, a similarly unusual Rab5-like GTPase of plants.

## Introduction

*Plasmodium falciparum* is a parasite that infects red blood cells (RBC) and causes severe human malaria, a disease that kills approximately 750,000 people per year, mostly children living in tropical Africa [1]. The malaria parasite spends much of its life cycle inside RBC, cells that provide it with an abundant food supply in the form of haemoglobin and a degree of protection from the host's immune system. For survival inside the RBC the parasite has to both import nutrients and to export metabolic waste products and like other eukaryotes it uses Rab GTPases to regulate vesicular trafficking [2] [3] [4].

Rabs are molecular switches belonging to the Ras-superfamily that are conserved from yeast to humans and regulate in space and time the budding and fusion of intracellular vesicles from donor to acceptor membranes [5]. Rabs vary in size from 20 to 29 kDa and were first identified in yeast and named Ypt proteins. Eleven Ypts were characterized in *Saccharomyces cerevisiae* and seven in *Schizosaccharomyces pombe* [6], whereas higher eukaryotes may possess more than 60 different Rabs [7]. Rab proteins exist in GTP-bound and GDP-bound states with switching from one state to another regulated by GTP Exchange Factors and GTPase Activating Proteins [8] [9]. GDP-bound Rabs are considered to be inactive and are associated with Rab GDP Dissociation Inhibitors (rabGDIs), whereas GTP-bound Rabs are considered to be active and able to recruit and interact with their effectors, which is a key specific Rab-mediated function [10]. Rabs from different species display a significant degree of sequence conservation in the GTP-binding domain, but they possess a variable N-terminal sequence responsible for recruitment of effectors and a hyper-variable C-terminal domain. The C-terminal domain confers the specific intracellular location for each Rab and has a motif containing one or two cysteines (CAAX, CC, CXC, CCX, CCXX) necessary for the isoprenylation by geranylgeranyl-transferases that is required for their association with vesicle membranes [11].

Mammalian cells encode three Rab5 isoforms (Rab5A, Rab5B and Rab5C) that share a high level of sequence identity and are involved in homotypic and heterotypic fusion of early and late endosomes [12] [13]. In spite of this homology different Rab5 isoforms can regulate separate functions, for example Rab5A and Rab5B, but not Rab5C, regulate the transfer of epidermal growth factor receptor from early to late endosomes [14]. siRNA knockdown of individual Rab5 isoforms demonstrated that collectively they organise early endosomes, late endosomes and lysosomes and can be considered master organisers of the endocytic system [15]. However, lineage-specific expansions of the gene family combined with functional diversification have contributed to species-specific variations in membrane trafficking. A classic example is the plant-specific Rab5-like GTPase called ARA6 that mediates trafficking from endosomes to the plasma membrane of *Arabidopsis thaliana*, where it contributes to the regulation of the plant's response to saline stress [16], [17], [18].

Multiple reciprocal BLAST analyses gave rise to the *P. falciparum* Rab (PfRab) nomenclature and identified a family of 11 PfRabs [19]. This family contains three Rab5 isoforms with PfRab5A (PF3D7_0211200) belonging to a restricted orthology group (OG4_36791) present only in *Apicomplexa* parasites known to invade erythrocytes (*Plasmodia*, *Babesia* and *Theileria*) [20]. However, PfRab5A has an insertion of 30 amino acids between the RabF1 and RabF2 motifs (Rab-effector binding motifs) [19] that is not observed in Rab5A of *Theileria* and *Babesia*, suggesting that some PfRab5A effectors might be *Plasmodium*-specific. Putative PfRab5A-effectors might be involved in the uptake of haemoglobin, as GFP-tagged PfRab5A decorates vesicles containing haemoglobin [21]. The PfRab5B orthology group (OG4_18709) is found more broadly; the C-terminus of PfRab5B (PF3D7_1310600) has no lipid-modification motif [19], [20], like human Rab8 and Rab23 [22] and ARA6 [18]. The PfRab5C (PF3D7_0106800) orthology group (OG4_10168) is the largest [20]. PfRab5C looks like a classical Rab5, but comparison of a 3D-model of PfRab5C with the known 3D-structure of mouse Rab5C showed that these two Rab5s present different amino acids at their effector-interaction surfaces, implying that PfRab5C has the potential to recruit parasite-specific effectors [19].

As *S. cerevisiae* possesses 11 Ypts/Rabs like *P. falciparum*, the two “Rabomes” were compared and a putative potential function was attributed to each PfRab [20]. In addition, potential PfRab functions have been inferred from over-expression of wild type and dominant-negative (GDP-on) mutants of *Toxoplasma gondii* (Tg) Rabs [23] [24] [25]. *Plasmodium* versus *Toxoplasma* comparison is only valid for true orthologues in the two *Apicomplexa* and is therefore not applicable to PfRab5A, but some potential *Plasmodium*-specific functions have been inferred from studies on the over-expression of wild type TgRab5B and a FKBPmyc-TgRab5C fusion [25]. Over-expressed TgRab5B was found to concentrate at endosomal-like compartments and to a lesser extent at the surface of *T. gondii*, whereas over-expression of TgRab5C variants blocked secretion of specific cargo to a subset of micronemes at the apex of the parasite [25]. Over-expression of both TgRab5B and TgRab5C FKBPmyc fusions was deleterious for *T. gondii* in culture.

The PfRab-interactome predicted that Casein Kinase 1 (PfCK1; PF3D7_1136500.1) is a specific PfRab5B-interacting protein and indeed, PfRab5B physically interacts with PfCK1 *in vitro* [20]. The subcellular distribution of PfCK1 has not yet been described [26], but in *T. gondii* CK1 is found both in the cytosol and at the plasma membrane [27]. We decided to test therefore, whether *in vivo* PfRab5B is competent to traffic PfCK1 to different subcellular locations in *P. falciparum*. Moreover, as PfRab5B lacks the necessary motif for isoprenylation, yet possesses recognisable myristoylation and palmitoylation motifs [19], we examined directly whether the N-terminal glycine residue of PfRab5B is a *bona fide* substrate for *P. falciparum* N-myristoyltransferase (NMT) [28] *in vitro* and whether *in vivo* its myristoylation is involved in targeting PfRab5B to specific subcellular membranes. Based on our results we propose that myristoylation (and palmitoylation) of PfRab5B allows it to traffic specific cargo to the parasite's plasma and food vacuole membranes.

## Results

### The N-terminal glycine residue in PfRab5B is required for N-myristoylation

**Figure 1:**
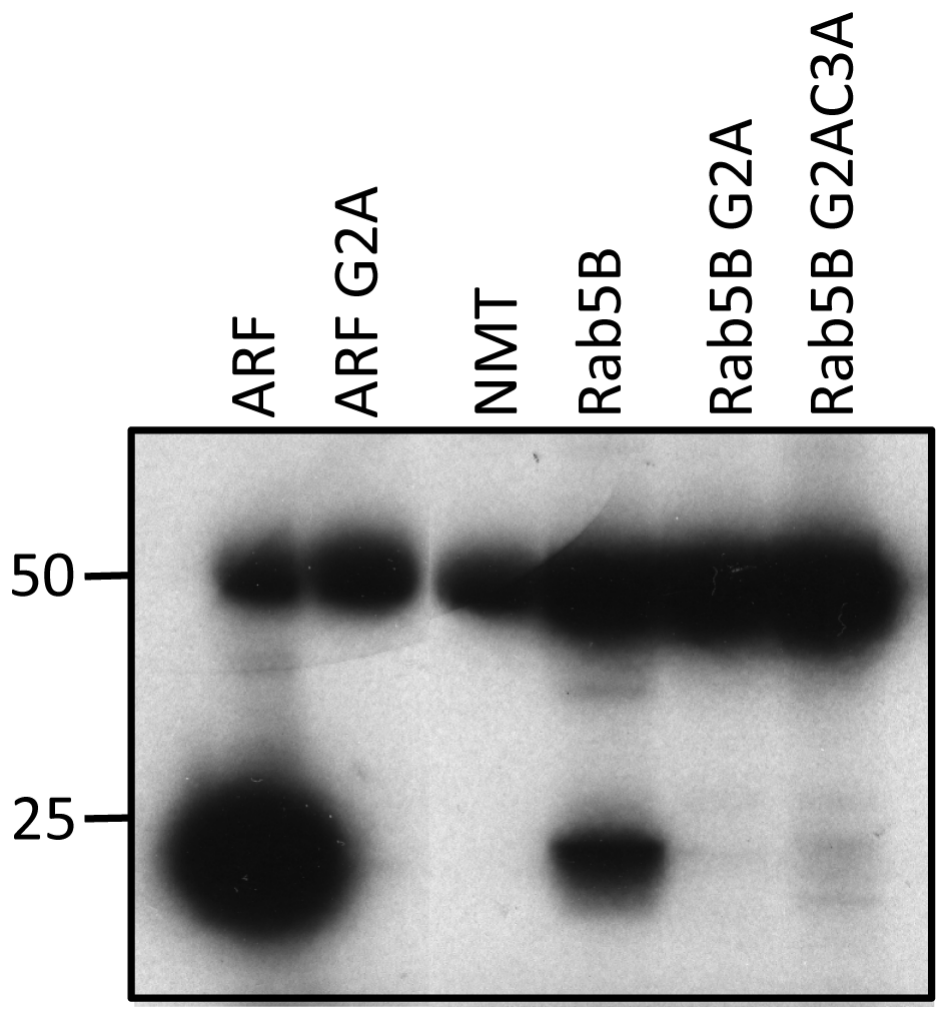
Recombinant PfRab5B is N-myristoylated by PfNMT *in vitro*. PfRab5B and the G2A and G2AC3A variants were incubated with [^3^H]-myristoyl CoA in the presence of N-myristoyl transferase. Recombinant ARF and ARFG2A were treated in the same way, acting as controls. N-myristoylation was detected by the incorporation of radiolabel into the substrate following SDS-PAGE and fluorography and the upper band at 50 kDa is due to label binding to PfNMT. Both PfRab5B and ARF migrated with mobility slightly faster than the 25 kDa molecular mass marker. No incorporation was detected if the N-terminal glycine residue of either protein was replaced with alanine (G2A).

To test the validity of the myristoylation motif prediction we produced recombinant PfRab5B protein and variants in which either the glycine or the glycine and cysteine residues at the N-terminus of PfRab5B (MGCSS) were changed to alanine. These His-tagged full-length recombinant wild type and variant PfRab5B proteins were purified and used as substrates in an N-myristoylation assay using recombinant *P. falciparum* N-myristoyl-transferase (PfNMT) [28] and tritiated myristoyl-CoA [29] (see Figure 1). Wild type and G2A-variant recombinant PfARF protein (PF3D7_1020900) were used as controls [30] [29]. The results showed that PfRab5B is a substrate for PfNMT (although the efficiency of myristoyl transfer was less than that for PfARF) and the presence of a glycine at position two is required for PfRab5B myristoylation *in vitro*, which occurs via peptide bond formation with the α-amino group of the N-terminal glycine. As expected, modification of both the myristoylation and the palmitoylation site (G2AC3A) had no additional effect.

### PfRab5B myristoylation in the parasite is essential for its targeting to membranes

**Figure 2:**
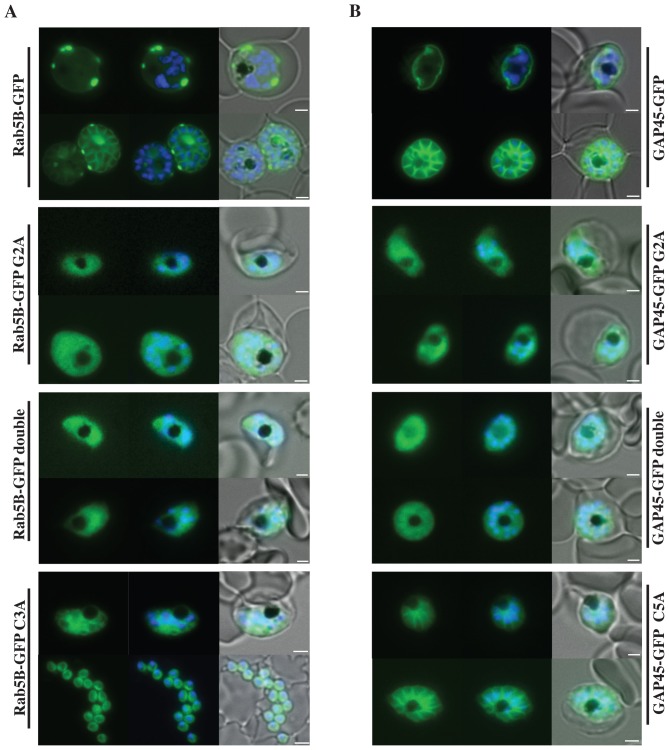
Localisation of PfRab5B-GFP fusion proteins in *P. falciparum*. Parasites were transfected with constructs expressing the N-terminal 28 amino acids of PfRab5B or 29 amino acids of PfGAP45 fused to GFP under a schizont stage-specific promoter (*msp3* 5′UTR region). Localisation of PfRab5B_28_-GFP (A) and GAP45_29_-GFP fusions (B) as well as myristoylation (G2A) and palmitoylation-deficient fusions (C3A in PfRab5B_28_-GFP and C5A in GAP45_29_-GFP) were investigated. The first image in a series corresponds to GFP fluorescence, the second a merge of GFP fluorescence with nuclear DAPI stain, and the third a merge of GFP fluorescence, DAPI and bright field images. Size bars are 2 µm.

We generated a transgenic *P. falciparum* line that expresses GFP fused to the N-terminal 28 amino acids of PfRab5B (see Table S1 in File S1). An episomally maintained plasmid was used for transfection, which expresses the fusion protein under the control of the *msp3* promoter [31] [32]. Asexual blood stages of this transgenic line were analysed by fluorescence microscopy. GFP-fluorescence was consistent with a location of the fusion protein in association with the parasite's plasma membrane (Figure 2, top left hand panel). As a positive control, we used a second transgenic *P. falciparum* line that expresses GFP fused to 29 amino acids from the N-terminus of glideosome associated protein (GAP)45 (Table S1 in File S1). GAP45 is known to be associated with the plasma membrane at its N-terminus [31]. In asexual blood stages of this transgenic parasite GFP was observed at the plasma membrane (Figure 2, top right hand panel). In transgenic parasites that expressed GFP fused to G2A variants of both PfRab5B and GAP45 the association of GFP with the plasma membrane was lost and we observed a diffuse GFP-staining throughout the cytoplasm of the parasites (Figure 2, second row panels). This cytoplasmic GFP-staining was also observed in blood stages expressing GFP-fusion proteins in which additional substitutions had been introduced (Table S1 in File S1): the C3A change in PfRab5B or C5A in GAP45 to give the double G+C variants (Figure 2, third row panels). The C to A variants alone allowed some membrane association (Figure 2, bottom row). Clearly, the N-terminal 28 or 29 amino acids in combination with myristoylation of G2 of both PfRab5B and GAP45, respectively, are sufficient to target the GFP-fusion proteins to the parasite's plasma membrane.

### PfRab5B-GFP and GAP45-GFP fusion proteins are embedded in membranes of *P. falciparum* parasites

**Figure 3:**
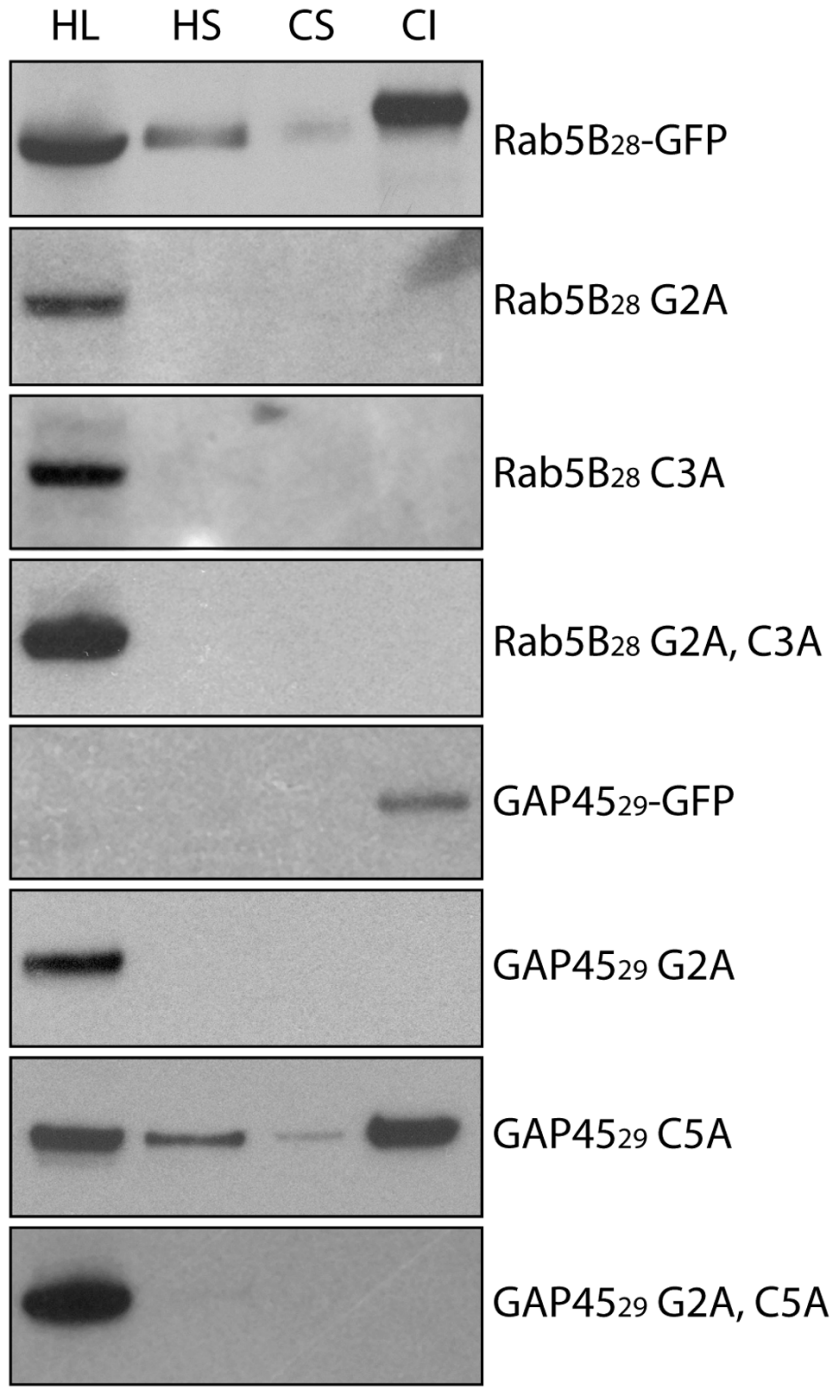
Subcellular fractionation of PfRab5B and GAP45 GFP chimeras. When fused to GFP, the first 28 amino acids of PfRab5B confer a partial membrane association to the fusion protein. PfRab5B_28_-GFP is found to varying degrees in the four fractions studied. Substitution of glycine at position two with alanine (PfRab5B_28_ G2A) results in loss of all membrane association, as does substitution of cysteine at position three (PfRab5B_28_ C3A). A known myristoylated and palmitoylated protein, GAP45_29_-GFP is found exclusively in membrane fractions and a G2A substitution results in an entirely soluble protein (GAP45_29_ G2A). A substitution of cysteine at position five causes a reduction in the proportion of protein associated with membranes (GAP45_29_ C5A). Double substitutions of glycine and cysteine result in a cytosolic protein for both PfRab5B_28_ and GAP45_29_ GFP fusion proteins. HL: hypotonic lysis buffer supernatant, HS: high salt buffer supernatant, CS: carbonate buffer supernatant, CI: carbonate buffer insoluble.

Schizonts of transgenic parasites expressing GFP fused to N-terminal sequences of PfRab5B and GAP45 (28 or 29 amino acids, respectively) were purified and submitted to subcellular fractionation using hypotonic and high salt buffers to release cytosolic proteins, followed by high pH carbonate extraction to fractionate peripheral membrane (carbonate soluble) and integral membrane (carbonate insoluble) proteins. The proteins in the four different fractions were resolved by SDS-PAGE and analysed by western blotting with an anti-GFP antibody (Figure 3). When the wild type PfRab5B N-terminal sequence was fused to GFP (PfRab5B_28_-GFP) the protein was found in all subcellular fractions (to differing degrees) indicating that not all of this fusion protein was associated with membranes despite the presence of both G2 and C3 residues. In contrast, both the GFP-fusion proteins containing the G2A and C3A variants were located solely in the hypotonic lysis soluble fraction, as was the variant with both substitutions (Figure 3, track 1). The GFP protein fused to the wild type GAP45 N-terminal sequence (GAP45_29_-GFP) was located exclusively in the carbonate-insoluble fraction (track 4) typical for a membrane-associated protein. Similar to the PfRab5B_28_G2A variant of the GFP fusion protein, GAP45_29_G2A was found exclusively in the soluble fraction consistent with myristoylation alone being necessary and sufficient for membrane association. In contrast, GAP45_29_C5A was distributed across the different fractions, suggesting that palmitoylation of the N-terminus of GAP45 contributes to its membrane association, but it is neither necessary, nor sufficient.

### Endogenous PfRab5B is associated with both the food vacuole and plasma membranes

**Figure 4:**
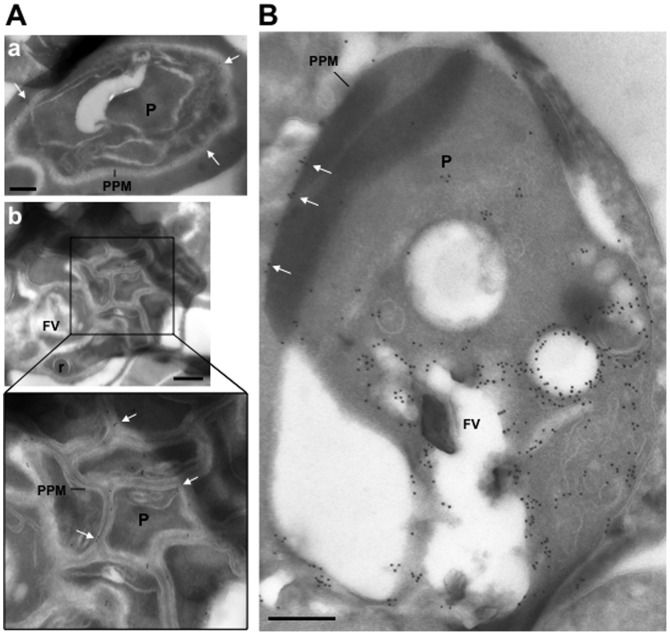
Ultrastructural detection of PfRab5b in *P. falciparum*-infected RBC. Immuno-electron microscopy of schizonts (A) and a large trophozoite (B) of *P. falciparum* labelled with specific anti-PfRab5B antibodies, revealing the presence of gold particles both on the food vacuole (FV) and the parasite plasma membrane (PPM, white arrows) in trophozoites and exclusively on parasite plasma membrane for late stages. P, parasite; r, rhoptry. Scale bars, 150 nm.

We generated antibodies to PfRab5B and verified their specificity by western blot (see Figure S1). These specific PfRab5B-specific antibodies were then used to determine the subcellular location of PfRab5B by immuno-electron microscopy. In Figure 4 micrographs are shown of two different stages of intraerythrocytic development, mature schizonts (Panel A) and a trophozoite (Panel B). Gold-labelled anti-PfRab5B antibodies decorated the parasite plasma membrane (PPM, white arrows) and were also observed associated with the food vacuole (FV) and neighbouring vesicles, as shown for the trophozoite in panel B. The distribution of endogenous PfRab5B was therefore similar to that of PfRab5B-GFP suggesting that the plasma- and food vacuole membrane associations are the result of myristoylation and/or palmitoylation at the N-terminus of PfRab5B.

### Endogenous PfRab5B colocalises with merozoite surface protein (MSP)1 and PfCK1, but not with haemoglobin

**Figure 5:**
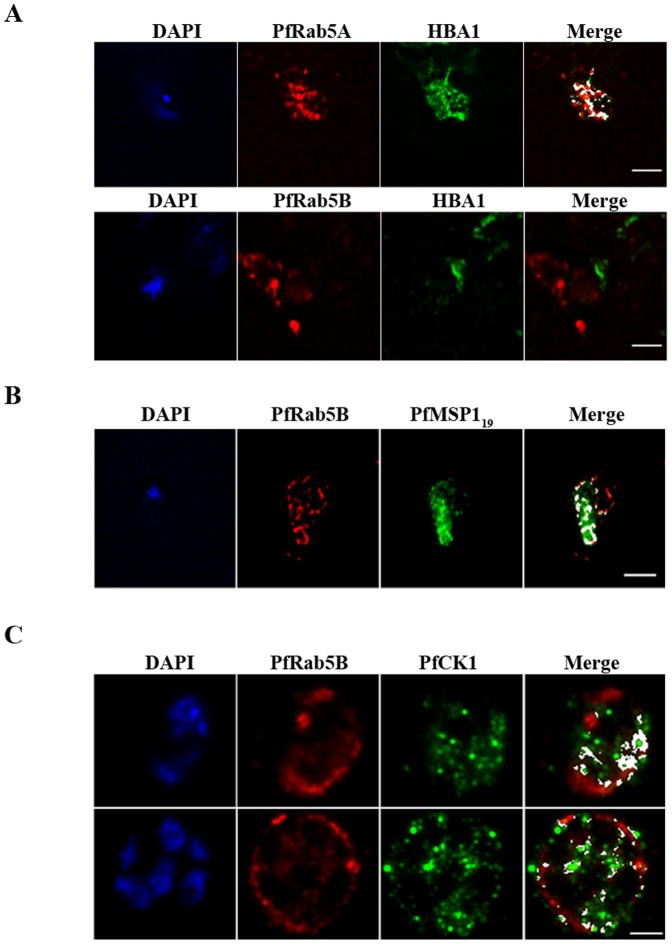
PfRab5B colocalises with PfMSP1 and PfCK1, but not with haemoglobin. (A) PfRab5A colocalises with haemoglobin (HBA1) containing vesicles (r = 0.724), unlike PfRab5B (r = 0.081; n = 3). (B) PfRab5B colocalises to differing degrees (r = 0.849; n = 3) with the C-terminal 19 kDa fragment of PfMSP1 on structures close to the food vacuole and the parasite nucleus shown in blue by DAPI staining. (C) PfRab5B colocalises with PfCK1 on intracellular structures (r = 0.615; n = 3) and at the parasite plasma membrane (r = 0.475; n = 3). Areas of colocalistaion are shown in white and used to calculate Pearson's r coefficients. Scale bars, 2 µm.

GFP-tagged PfRab5A has been described as associated with vesicles harbouring haemoglobin imported by the parasite from the erythrocyte cytosol [21]. The association of PfRab5B with the food vacuole membrane and adjacent vesicles led us, therefore, to look at an association with haemoglobin-containing structures using indirect immunofluorescence. Specific anti-PfRab5A antibodies (see Figure S1) were used as a positive control. In contrast to PfRab5A (r = 0.724; n = 3) we could find no evidence for co-localisation between PfRab5B and haemoglobin (HBA1, r = 0.081; n = 3) in trophozoites, the developmental stage that imports haemoglobin (Figure 5, panel A).

Following merozoite invasion, a C-terminal fragment of MSP1 is associated with the developing food vacuole membrane [33], a localisation that is similar to that observed for PfRab5B, as shown in Figure 4. We confirmed therefore that antibodies to MSP1 and PfRab5B significantly (r = 0.849; n = 3) decorate the same structure (Figure 5, panel B).

As PfRab5B was found to physically interact with PfCK1 *in vitro* [20] we looked for evidence that PfRab5B-positive vesicles might traffic the kinase to different subcellular locations. Depending on the developmental stage of the parasite (indicated by the number and size of DAPI-stained nuclei; Figure 5, panel C) the degree of association between PfRab5B and PfCK1 varied (r = 0.615; n = 3 in late schizonts and r = 0.475; n = 3 in young schizonts). Clearly, PfRab5B-positive vesicles can traffic more than one type of cargo, however the failure to observe any association of PfRab5B with haemoglobin suggests that PfRab5B and PfRab5A perform separate trafficking functions.

### *Plasmodium* Rab5B is essential for asexual blood stage parasite development

Over-expression of individual Rab5s and/or Rab5 GDP-on variants in transgenic cells has the potential to generate phenotypes due to sequestering effector proteins that are shared by the different Rab5 isoforms, and therefore the use of such ‘over-expressing’ transgenic cells may hamper discrimination between the different functions of the Rab isoforms. Therefore, we decided to analyse the function of *Plasmodium rab5b* by attempting to switch off its expression using targeted gene-deletion by reverse genetics. We targeted the orthologous *rab5b* gene of the rodent malaria parasite *P. berghei* for gene deletion using standardized transfection methods [34]. In multiple experiments (Figure S2 in File S1) we were unable to select for mutants lacking the *Pbrab5b* gene indicating that PbRab5B has an essential function during asexual blood stage growth and multiplication. Moreover, these results also indicate that PbRab5A and PbRab5C cannot compensate for the function of PbRab5B.

## Discussion

*P. falciparum* has a small family of 11 PfRabs and all but PfRab5B possess classical geranyl-geranylation motifs at the C-terminus. This isoprenyl modification is present in 10 of the PfRabs presumably anchoring these GTPases to membrane. In contrast, we have shown here that PfRab5B is myristoylated allowing membrane attachment at the N-terminal glycine. PfRab5B peptides have recently been detected in a global *in vivo* myristome of *P. falciparum* [35]. Our inability to knock out the *rab5b* gene of *P. berghei* indicates that *Plasmodium* Rab5B performs functions essential for parasite asexual blood stage development. Potential functions may include trafficking MSP1-tagged vesicles from the plasma membrane to form the food vacuole membrane following RBC invasion [33] and PfCK1 to the plasma membrane. However, in contrast to PfRab5A [21] we never observed PfRab5B associated with haemoglobin-positive structures indicating that these two *Plasmodium*-specific Rab5 isoforms perform separate functions.

The presence of the wild type PfRab5B-GFP fusion protein in different subcellular fractions suggests that endogenous PfRab5B associates with varying affinity to membranes. However, as the wild type PfRab5B-GFP fusion protein was also found in the soluble fraction the results suggest that not all of the endogenous PfRab5B is myristoylated and/or palmitoylated at a given time. Substitution of the N-terminal glycine with alanine abolished the association of the PfRab5B-GFP fusion protein with membrane, clearly demonstrating that myristoylation of the PfRab5B N-terminal amino acid sequence is necessary and sufficient to attach it to membranes. Although we did not directly examine the palmitoylation status of PfRab5B substituting a cysteine with an alanine ablated membrane-targeting of the GFP-fusion protein as judged by solubility analysis, although some apparent association with the plasma membrane was detected by live cell imaging. Palmitoylation of endogenous PfRab5B therefore, seems to contribute to its subcellular location and indeed, a recent analysis of the *P. falciparum* palmitoylation proteome identified PfRab5B, as a component [36], [37]. Protein palmitoylation is reversible and dynamic and can result in proteins targeted to specific membrane domains [38].

PfRab5B resembles ARA6 a Rab5-like GTPase considered unique to plants such as *A. thaliana* [16], [17], [18]. When ARA6 was fused to GFP and expressed in protoplasts prepared from cultured *Arabidopsis* cells, both punctate and plasma membrane localisation was observed [18]. Interestingly, when the N+3 cysteine was changed to serine leaving the N+2 glycine intact ARA6 became uniquely myristoylated and the GFP-fusion no longer localised to endosomes, indicating that additional palmitoylation is necessary for endosome targeting [18]. When the N-terminal glycine was changed to an alanine ARA6-GFP was found in the cytosol demonstrating that myristoylation is essential for membrane-association, as we have found for PfRab5B here. In addition, the C-terminal domain of Ara6 also plays a role in determining its subcellular localisation [18] and it is possible that the C-terminus of PfRab5B plays a similar role. We did not observe PfRab5B_28_-GFP associated with the food vacuole membrane (Fig. 2), unlike endogenous PfRab5B (Fig. 4), and as only the first N-terminal 28 amino acids of PfRab5B are present in the GFP-chimera this implies that the C-terminus of PfRab5B might contribute to its food vacuole location. However, unlike PfRab5B, palmitoylation of ARA6 is not required for membrane association *per se*, only for endosome-specific targeting. It is possible that additional targeting information is encoded in the N-terminal amino acid sequence of myristoylated and palmitoylated proteins, since the subcellular fractionation of PfRab5B-GFP and GAP45-GFP fusion proteins was not identical following replacement of the palmitoylated cysteine residue. For PfRab5B with the C3A substitution the fusion protein was found only in the soluble fraction, in contrast to GAP45 with the C5A substitution, for which the fusion protein was distributed across all subcellular fractions. The N-terminal sequence of GAP45 fused to GFP has ten basic and six acidic residues, whereas the N-terminal sequence of PfRab5B fused to GFP has only four basic and one acidic residue, so the GAP45 N-terminal sequence is much more highly charged and this greater charge might contribute to greater affinity for membrane phospholipid head groups.

ARA6 acts in an endosomal trafficking pathway by modulating the assembly of a distinct SNARE complex that includes VAMP727 [16]. A family of *P. falciparum* SNAREs has been described [39] [40] and PfVAMP8 (PF3D7_1303200.1) displays the highest identity (5.6e-35) to VAMP727 suggesting that perhaps PfRab5B regulates trafficking via assembly of PfVAMP8 complex. Whether a *P. falciparum* PfRab5B/PfVAMP8 complex is involved in regulating a stress response, as is the ARA6/VAM727 complex in *Arabidopsis* [17] is an interesting and open question. Unlike PfRab5A we never observed PfRab5B associated with haemoglobin containing vesicles, implying that haemoglobin uptake would likely not involve the PfVAMP8 complex. Clearly, in malaria causing parasites in spite of having a small Rab family of only 11 GTPases, PfRab5A, PfRab5B and PfRab5C isoforms regulate independent and crucial functions.

## Materials and Methods

### Attempts to generate *P. berghei rab5b* gene deletion mutants

Female Swiss OF1 mice (6–8 weeks old; Charles River/Janvier) were used. All mouse experiments received approval from the Animal Experiments Committee at the Medical Centre of Leiden University (DEC 07171; DEC 10099). The Dutch Experiments on Animal Act is established under European guidelines (EU directive no. 86/609/EEC regarding the Protection of Animals used for Experimental and Other Scientific Purposes). In order to create *P. berghei rab5b* (PBANKA_140910) gene deletion mutants, a DNA construct pL1709 was generated that would target *Pbrab5b* by double crossover homologous recombination (Figure S1), as previously described [34]. The targeting plasmid was made to target both the 5′ and 3′ regions of *Pbrab5b* gene and contains the drug selectable marker human *dhfr* (*hdhfr*) (See Table S2 in File S1 for details of the primers). Prior to transfection, the plasmid was linearized by digestion with enzymes *Hind*III and *Eco*RI. The *P. berghei* ANKA reference reporter parasite line *Pb*GFP-Luc_con_ of the ANKA strain of *P. berghei* was used for two independent transfection experiments (See RMgm-29 in www.pberghei.eu for details of the *Pb*GFP-Luc_con_ line). All experimental procedures including animal work and transfection and selection protocols to generate gene knock-out parasites were performed as described [34].

### Expression constructs and purification of recombinant proteins

To make C-terminal-tagged PfRab5A-hexaHis and PfRab5B-hexaHis constructs for expression in *Escherichia coli*, the CDS of *Pfrab5a* (PF3D7_0211200) and *Pfrab5b* (PF3D7_1310600) were PCR amplified from *P. falciparum* 3D7 cDNA and cloned into *BamHI/XhoI* sites of the *E. coli* expression vector pET-21d (Novagen, EMD4Biosciences). The sequences of all expression constructs were verified.

*Pfrab5a* and *Pfrab5b* constructs were transformed into BL21-CodonPlus (DE3)–RIL strain (Stratagene). BL21 cells were grown at 37°C in LB medium containing 34 µg/ml chloramphenicol and 100 µg/ml ampicillin to an absorbance of approximately 0.6 at 600 nm. Protein expression was induced by adding 0.2 mM IPTG, and the cultures were incubated overnight at 20°C. Cells were harvested by centrifugation at 5000 *g* for 20 min and re-suspended in 6 M urea buffer supplemented with protease inhibitor cocktail (Roche), then stored at −80°C. The His-tagged proteins were purified on Ni-NTA agarose (Qiagen).

To generate *Pfrab5B^G2A^*, *Pfrab5B^C3A^* and *Pfrab5B^G2AC3A^* mutations, PCR site-directed mutagenesis was performed using *Pfrab5b* cloned into pET-21d vector as a template. Thereafter, PCR products were incubated at 37°C with *DpnI* enzyme for an hour. The sequences of all expression constructs were verified.

### Specific antibodies

Specific PfRab5A antibodies were raised in rabbits and specific anti-PfRab5B antibodies were raised in rats (Eurogentec) to full-length His-tagged recombinant PfRab5A and PfRab5B (see above). Specific anti-PfCK1 antibodies were a gift of Dominique Dorin-Semblat and Christian Doerig and anti-MSP1_19_ antibodies have been described [41].

### *In vitro* N-myristoylation assay

The *in vitro* myristoylation assay was performed essentially as described previously [29] using bacterially expressed recombinant PfNMT, recombinant substrate protein and [^3^H]myristoyl-CoA. Recombinant PfARF and G2A PfARF were used as controls. 0.5 µg PfNMT was incubated with either 5 µg PfRab5B or 2.5 µg ARF proteins and myristoyl-CoA (0.007 MBq) for 2 h at room temperature in reaction buffer (30 mM Tris-Cl, pH 6.8, 0.5 mM EGTA, 0.45 mM EDTA, 4.5 mM 2-Mercaptoethanol, 1% Triton X-100). Fractions from each reaction were resolved by SDS-PAGE under reducing conditions on precast 12% NuPAGE acrylamide gels. The gels were treated for fluorography, dried and exposed to X-ray film to detect the tritium label.

### Parasite culture

*P. falciparum* 3D7 was maintained in human O+ erythrocytes using RPMI 1640 medium supplemented with 1% Albumax at 3% hematocrit in gassed (90% nitrogen, 5% oxygen, 5% carbon dioxide) in flasks at 37°C. Parasites were synchronized using Percoll gradient centrifugation.

### Plasmid constructs and transfection of *P. falciparum*

To generate GFP-fusion protein expressing parasites we used primers to amplify the sequence encoding the N-terminal fragments of PfRab5B and GAP45, and inserting nucleotide changes to introduce alanine instead of glycine or cysteine as indicated in Table S1 in File S1. The PCR reactions were purified using a QIAGEN quick purification kit and digested with *Sac*II and *Avr*II. The digested inserts were gel extracted then ligated with digested pHH3 vector distal to the *msp3* promoter [31]. Resulting plasmids were transformed into DH5α and the sequence of each construct was established using DNA sequencing (Geneservice). 100 µg of sterile plasmid DNA in 100 mM Tris-HCl, 10 mM EDTA, pH 8.0 buffer was transfected by electroporation into parasites at 10% ring stage parasitaemia and culture was continued in the presence of blasticidin (2.5 µg/ml).

### Subcellular fractionation of schizonts and western blotting

The method described previously [32] was used with minor modifications; all incubations were carried out at 4°C and the buffers contained complete protease inhibitor cocktail (Roche). Parasite pellets were solubilised in 10 times the volume of hypotonic lysis buffer (10 mM Tris, 5 mM EDTA, pH 8.0) followed by centrifugation at 100,000 *g* for 30 min at 4°C and recovery of the supernatant. The pellet was washed once by repetition of the process and subsequently extracted with high-salt buffer (10 mM Tris, 5 mM EDTA, 500 mM NaCl, pH 7.5), followed by centrifugation. The supernatant was retained and the pellet was extracted again in carbonate buffer (100 mM sodium carbonate, pH 11.0). Following a final centrifugation the three supernatants and the remaining carbonate-insoluble material were each mixed with SDS sample loading buffer and subjected to SDS-PAGE and western blotting with anti-GFP antibodies. Samples were heated at 95°C for 5 min prior to being separated by SDS-PAGE on a pre-cast 12% Bis-Tris NuPAGE polyacrylamide gel (Invitrogen) and transferred to nitrocellulose membrane according to standard protocols. Blots were blocked in a solution of 5% w/v of non-fat milk powder and 0.2% Tween20 in PBS for 1 h then probed with a mouse anti-GFP antibody (Roche) diluted in blocking solution for 1 h. Bound antibody was detected by incubation with a horseradish peroxidase conjugated anti-mouse secondary antibody (Biorad) and visualised using enhanced chemiluminescence western blotting detection reagents (GE Healthcare).

### Fluorescence Microscopy

Live synchronized parasite populations expressing GFP-tagged proteins were examined by epifluorescence microscopy on an Axioplan 2 microscope (Zeiss), equipped with a Plan Apochromat 100×/1.4 oil immersion objective and an AxioCam HRc camera [31]. Parasites were labelled with DAPI DNA stain, then a cell suspension was placed on a slide and overlaid with a Vaseline rimmed cover slip. Parasitized erythrocytes were viewed live, and the dual colour fluorescence images were captured using Axiovision 4.6.3 software and edited using Adobe Photoshop CS4.

For indirect immunofluorescence smears of red blood cells infected with *P. falciparum* 3D7 strain were fixed for 5 min using 100% cold methanol. In the case of haemoglobin labelling, cells were pre-treated with saponin prior to fixation. Cells were washed with PBS, and then permeabilized with 0.1% Triton X100 in PBS for 5 min. After washing with PBS, slides were blocked in PBS containing 3%(w/v) BSA for 1 h at room temperature. The slides were incubated successively for 1 h with primary antibodies, depending on the co-labelling desired: mouse anti-haemoglobin (1∶500, Sigma), rat anti-PfRab5B (1∶200), rabbit anti-PfRab5A (1∶500), mouse anti-MSP-1_19_ (1∶500), and rabbit anti-PfCK1 (1∶500). The slides were washed four times and incubated with secondary antibodies depending on the primary antibodies used: AlexaFluor 594 anti-rat IgG antibody (1∶3000, Molecular Probes), AlexaFluor 488 anti-mouse IgG antibody (1∶2000, Molecular Probes), AlexaFluor 488 anti-rabbit IgG antibody (1∶5000, Molecular Probes), and with DAPI (1 µg/ml). Then, the slides were washed and mounted (Dako) and examined under a microscope (Leica DMI 6000, ×100 objective, NA 1.4 oil) with a cooled charge-coupled device camera (Micromax). Z-stack images were acquired with MetaMorph (Universal Imaging) and de-convoluted with Huygens (SVI). Images were analyzed and processed with ImageJ (NIH) and Photoshop (Adobe Systems Inc). For the merge, the ImageJ co-localization plug-in was used. Pearson's coefficient of co-localization was attributed with the ImageJ JACoP plug-in [42].

### Immuno-electron microscopy

*P. falciparum*-infected red blood cells (mixed development stages) were fixed and sectioned as described [43]. The sections were immuno-labelled with anti-PfRab5B antibody at 1/10 dilution in PBS/1% fish skin gelatin, then incubated with anti-IgG antibodies, followed directly by 10 nm protein A-gold particles before examination with a Philips CM120 Electron Microscope (Eindhoven, the Netherlands) at 80 kV.

## Supporting Information

File S1 **Supporting Information contains Supplementary Tables S1 and S2 and legends for Supplementary Figures S1 and S2.** Table S1: N-terminal sequences of PfRab5B, GAP45, and their variants used in the study. Under “Construct name” are listed all the different wild type and mutant GFP chimera expression plasmids used in the study. The subscript number corresponds to the number of amino acids fused to GFP. Under “Sequence fused to GFP” are the different wild type and mutant amino acids (underlined) sequences fused to the N-terminus of GFP. Table S2: Primers used for generation of the gene-deletion construct and for genotyping parasites. Listed in the number of each primer, its sequence with the restriction site underlined, its description and the name of the restriction enzyme. Figure S1: Specificity of anti-PfRab5A and PfRab5B antibodies. Different amounts of purified His-tagged PfRabs recombinant proteins were separated using a 15% polyacrylamide gel and transferred to a nitrocellulose membrane. (**A**) The blot was first incubated with the rabbit anti-PfRab5A (1∶500) and then incubated with an anti-rabbit peroxidase-conjugated secondary antibody (1∶15000, Sigma Aldrich). The lower panel shows protein loading by Ponceau S staining. (**B**) The blot was first incubated with the rat anti-PfRab5B antibody (1∶1000) and then with an anti-rat peroxidase-conjugated secondary antibody (1∶4000, Sigma Aldrich). The lower panel shows protein loading by Ponceau S staining. Each anti-PfRab5 antibody specifically reacted only with its corresponding recombinant protein. Figure S2: Unsuccessful attempts to generate *P. berghei rab5b* (PBANKA_140910) gene-deletion mutants. (A) Schematic representation of the gene-deletion construct used for targeting the *rab5b* gene for deletion and the expected gene locus before and after disruption. The construct that has *hdhfr* as a drug selectable marker (SM, black) is designed to disrupt the open reading frame (ORF) of the *Pbrab5b* genes by double crossover homologous recombination. The expected genomic integration of the construct into the *P. berghei* genome is indicated, and the size and location of the *Pbrab5b* targeting regions (hatched boxes) are shown in relation to the *Pbrab5b* gene ORF. The targeting regions are indicated as +/− bp distance from the putative start codon. The location and name of the primers used for diagnostic PCR are shown. (B) Diagnostic PCR of genomic DNA of parasites selected after transfection with gene-deletion construct pL1709 (see A) showing that *rab5b* ORF was not disrupted in the selected parasites. Two independent transfection experiments were performed and the parasites that survived drug selection with pyrimethamine contained both the selectable marker and the intact ORF. The following primers were used: 5′ integration (5′): 6909/3189; 3′ integration: (3) 4592/6910; amplification of the *hdhfr* cassette (M): 307C/3187; ORF (O): 6911/6912. Genomic DNA of wild type *P. berghei* parasites (wt) was used as control. Two faint non-specific bands are amplified with the 3′-primers.
(DOCX)
