# Correction: *Plasmodium falciparum* Rab5B Is an N-Terminally Myristoylated Rab GTPase That Is Targeted to the Parasite's Plasma and Food Vacuole Membranes

**DOI:** 10.1371/journal.pone.0096592

**Published:** 2014-04-24

**Authors:** The PLOS ONE Staff

Figure 2 and Figure 4 do not appear in the PDF version of this article. The publisher apologizes for the errors. Please see Figures 2 and 4 here or in the online version of the article.

**Figure 2:**
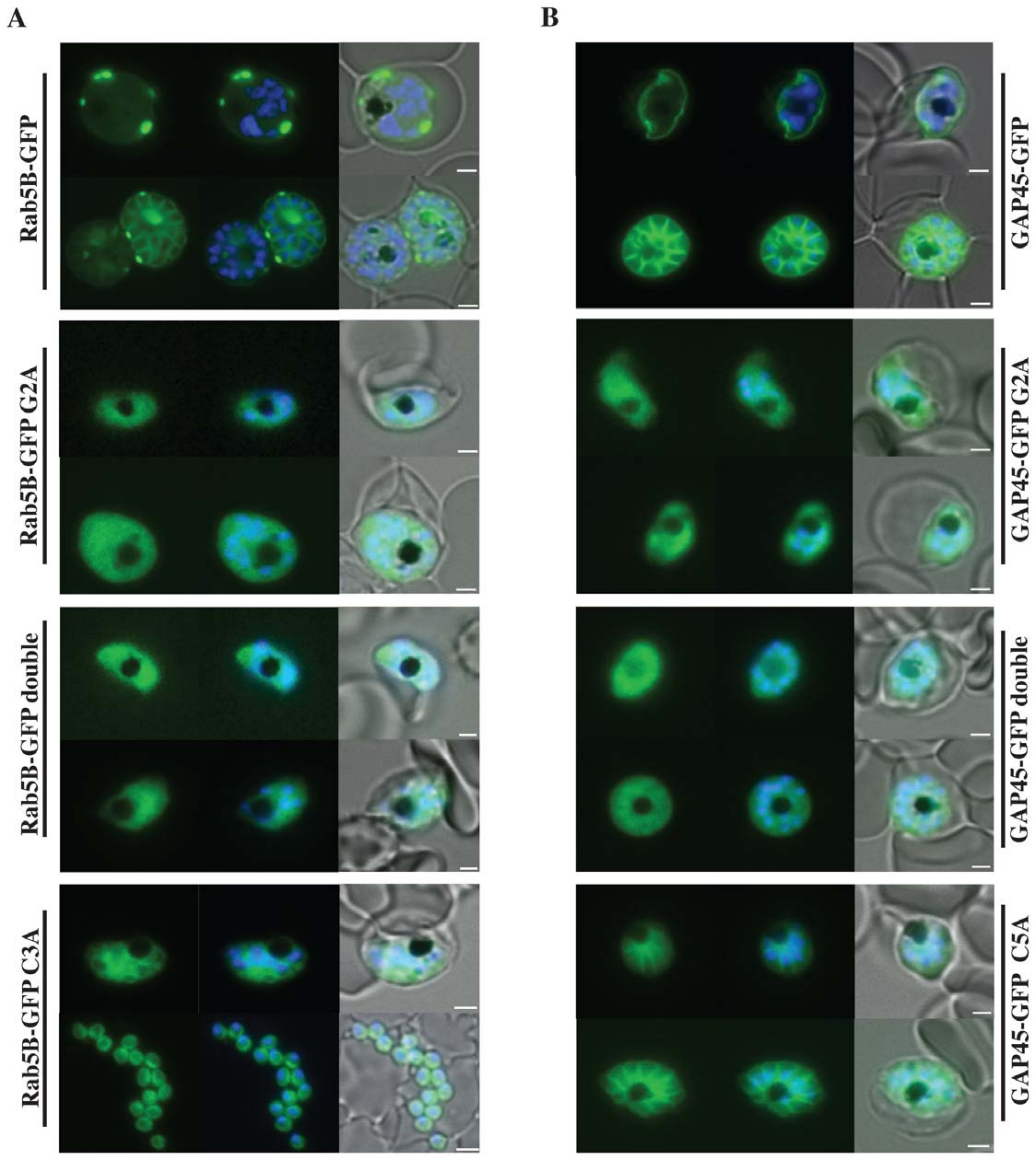
Localisation of PfRab5B-GFP fusion proteins in *P. falciparum*. Parasites were transfected with constructs expressing the N-terminal 28 amino acids of PfRab5B or 29 amino acids of PfGAP45 fused to GFP under a schizont stage-specific promoter (*msp3* 5′UTR region). Localisation of PfRab5B_28_-GFP (A) and GAP45_29_-GFP fusions (B) as well as myristoylation (G2A) and palmitoylation-deficient fusions (C3A in PfRab5B_28_-GFP and C5A in GAP45_29_-GFP) were investigated. The first image in a series corresponds to GFP fluorescence, the second a merge of GFP fluorescence with nuclear DAPI stain, and the third a merge of GFP fluorescence, DAPI and bright field images. Size bars are 2 µm.

**Figure 4:**
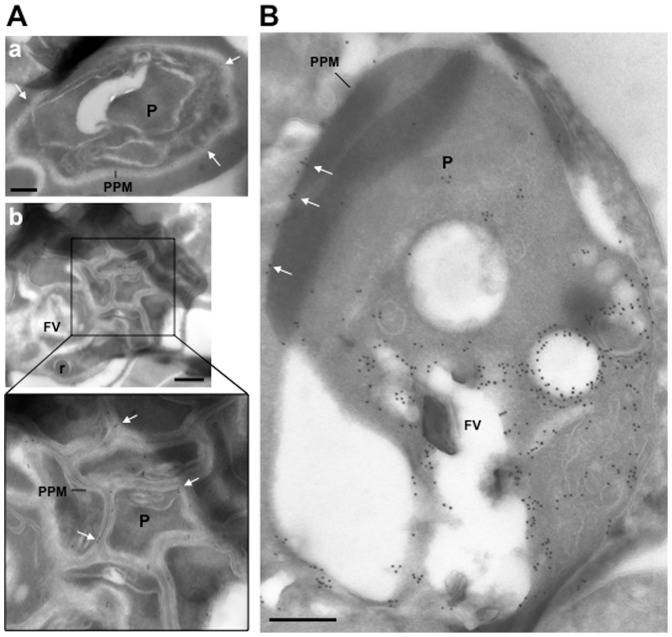
Ultrastructural detection of PfRab5b in *P. falciparum*-infected RBC. Immuno-electron microscopy of schizonts (A) and a large trophozoite (B) of *P. falciparum* labelled with specific anti-PfRab5B antibodies, revealing the presence of gold particles both on the food vacuole (FV) and the parasite plasma membrane (PPM, white arrows) in trophozoites and exclusively on parasite plasma membrane for late stages. P, parasite; r, rhoptry. Scale bars, 150 nm.
