# Supplementary material for: Plasmodium falciparum Rab5B Is an N-Terminally Myristoylated Rab GTPase That Is Targeted to the Parasite's Plasma and Food Vacuole Membranes
Source: PLoS One. 2014 Feb 3;9(2):e87695. doi: 10.1371/journal.pone.0087695 (PMC3912013; doi:10.1371/journal.pone.0087695)
Supplement: File S1 — Supporting Information contains Supplementary Tables S1 and S2 and legends for Supplementary Figures S1 and S2. Table S1: N-terminal sequences of PfRab5B, GAP45, and their variants used in the study. Under “Construct name” are listed all the different wild type and mutant GFP chimera expression plasmids used in the study. The subscript number corresponds to the number of amino acids fused to GFP. Under “Sequence fused to GFP” are the different wild type and mutant amino acids (underlined) sequences fused to the N-terminus of GFP. Table S2: Primers used for generation of the gene-deletion construct and for genotyping parasites. Listed in the number of each primer, its sequence with the restriction site underlined, its description and the name of the restriction enzyme. Figure S1: Specificity of anti-PfRab5A and PfRab5B antibodies. Different amounts of purified His-tagged PfRabs recombinant proteins were separated using a 15% polyacrylamide gel and transferred to a nitrocellulose membrane. (A) The blot was first incubated with the rabbit anti-PfRab5A (1∶500) and then incubated with an anti-rabbit peroxidase-conjugated secondary antibody (1∶15000, Sigma Aldrich). The lower panel shows protein loading by Ponceau S staining. (B) The blot was first incubated with the rat anti-PfRab5B antibody (1∶1000) and then with an anti-rat peroxidase-conjugated secondary antibody (1∶4000, Sigma Aldrich). The lower panel shows protein loading by Ponceau S staining. Each anti-PfRab5 antibody specifically reacted only with its corresponding recombinant protein. Figure S2: Unsuccessful attempts to generate P. berghei rab5b (PBANKA_140910) gene-deletion mutants. (A) Schematic representation of the gene-deletion construct used for targeting the rab5b gene for deletion and the expected gene locus before and after disruption. The construct that has hdhfr as a drug selectable marker (SM, black) is designed to disrupt the open reading frame (ORF) of the Pbrab5b genes by double crossover homolog [file pone.0087695.s001.docx]

**Table S1. N-terminal sequences of PfRab5B, GAP45 and their variants used in the study**

| **Construct name** | **Sequence fused to GFP** |
| --- | --- |
| PfRab5B_28_ | M**G**CSSSTERLTSTKNINIVTSPAQQQKK |
| PfRab5B_28_G2A | M**A**CSSSTERLTSTKNINIVTSPAQQQKK |
| PfRab5B_28_C3A | MG**A**SSSTERLTSTKNINIVTSPAQQQKK |
| PfRab5B_28_G2A, C3A | M**AA**SSSTERLTSTKNINIVTSPAQQQKK |
| GAP45_29_ | M**G**NK**C**SRSKVKEPKRKDIDELAERENLKK |
| GAP45_29_G2A | M**A**NKCSRSKVKEPKRKDIDELAERENLKK |
| GAP45_29_C5A | MGNK**A**SRSKVKEPKRKDIDELAERENLKK |
| GAP45_29_G2A, C5A | M**A**NK**A**SRSKVKEPKRKDIDELAERENLKK |

**Table S2. Primers used for generation of the gene-deletion construct and for genotyping parasites**

| **No.** | **Primer sequences (restriction site)** | **Description** | **Restriction site** |
| --- | --- | --- | --- |

*Primers for generation of the gene-deletion construct*

| 5'F  5'R  3'F  3'R | GTATAATATTGTATAAGCTTAATATATGG CAAAAAAGCTGCTCCTGCAGTTAC GGTACCACGCCGTAATAATAA  TTATGTGAATTCAATATCGATTTGC |  | *Hind*III  *Pst*I  *Kpn*I  *Eco*RI |
| --- | --- | --- | --- |
| *Primers for PCR analyses* | | | |
| 6909 | TTAAAATTGTTAGTTGCTTTGTG | *rab5b* 5’in-F |  |
| 6910 | TATGCCAAATTTAATAGAAAATTCAG | *rab5b* 3’in-R |  |
| 6911 | GCAGCTTTTTTGCACCATAC | *rab5b*ORF-F |  |
| 6912 | TTACCTCTGAATTTATTTTTTGTG | *rab5b*ORF-R |  |
| 307C | GCTTAATTCTTTTCGAGCTC | *eef1a* F |  |
| 3187 | GTGTCACTTTCAAAGTCTTGC | h*dfhr* R |  |
| 3189 | CTGGTGCTTTGAGGGGTG | *eef1a* R |  |
| 4592 | GGTAAGAAGACCTGGTTCTC | h*dhfr* F |  |

**Figure S1: Specificity of anti-PfRab5A and PfRab5B antibodies.**

Different amounts of purified His-tagged PfRabs recombinant proteins were separated using a 15% polyacrylamide gel and transferred to a nitrocellulose membrane. (A**)** The blot was first incubated with the rabbit anti-PfRab5A (1:500) and then incubated with an anti-rabbit peroxidase-conjugated secondary antibody (1:15000, Sigma Aldrich). The lower panel shows protein loading by Ponceau S staining. (B**)** The blot was first incubated with the rat anti-PfRab5B antibody (1:1000) and then with an anti-rat peroxidase-conjugated secondary antibody (1:4000, Sigma Aldrich). The lower panel shows protein loading by Ponceau S staining. Each anti-PfRab5 antibody specifically reacted only with its corresponding recombinant protein.

**Figure S2: Unsuccessful attempts to generate *P. berghei rab5b* (PBANKA_140910) gene-deletion mutants.**

(A) Schematic representation of the gene-deletion construct used for targeting the *rab5b* gene for deletion and the expected gene locus before and after disruption. The construct that has *hdhfr* as a drug selectable marker (SM, black) is designed to disrupt the open reading frame (ORF) of the *Pbrab5b* genes by double crossover homologous recombination. The expected genomic integration of the construct into the *P. berghei* genome is indicated, and the size and location of the *Pbrab5b* targeting regions (hatched boxes) are shown in relation to the *Pbrab5b* gene ORF. The targeting regions are indicated as +/- bp distance from the putative start codon. The location and name of the primers used for diagnostic PCR are shown. (B) Diagnostic PCR of genomic DNA of parasites selected after transfection with gene-deletion construct pL1709 (see A) showing that *rab5b* ORF was not disrupted in the selected parasites. Two independent transfection experiments were performed and the parasites that survived drug selection with pyrimethamine contained both the selectable marker and the intact ORF. The following primers were used: 5' integration (5’): 6909/3189; 3' integration: (3) 4592/6910; amplification of the *hdhfr* cassette (M): 307C/3187; ORF (O): 6911/6912. Genomic DNA of wild type *P. berghei* parasites (wt) was used as control. Two faint non-specific bands are amplified with the 3’-primers.
